# Two Symptoms Strongly Suggest Benign Paroxysmal Positional Vertigo in a Dizzy Patient

**DOI:** 10.3389/fneur.2020.625776

**Published:** 2021-01-14

**Authors:** Victor S. van Dam, Britta D. P. J. Maas, Tjard R. Schermer, Peter-Paul G. van Benthem, Tjasse D. Bruintjes

**Affiliations:** ^1^Apeldoorn Dizziness Centre, Gelre Hospital, Apeldoorn, Netherlands; ^2^Department of Otorhinolaryngology and Head and Neck Surgery, Leiden University Medical Centre, Leiden, Netherlands

**Keywords:** BPPV, patient history, symptoms, diagnosis, dizziness, vertigo, predictive model

## Abstract

**Introduction:** This prospective cohort study determined which questions in patient history are most likely to identify symptoms that are independently associated with a diagnosis of benign paroxysmal positional dizziness (BPPV) in patients presenting with dizziness, and to evaluate whether the patient's age and type of BPPV are of influence.

**Methods:** We included adult patients with dizziness referred to our dizziness center, Apeldoorn, the Netherlands, from December 2018 to November 2019. All patients completed a questionnaire, underwent vestibular testing and received a diagnosis. Symptoms strongly suggesting BPPV were tested with multivariable analysis to determine their independent associations with BPPV. Subgroup analysis was performed for patient age, and the type of BPPV.

**Results:** We included a total of 885 patients, 113 of whom (13%) were diagnosed with BPPV. The duration of dizziness spells <1 min (Q2) and dizziness provoked by rolling over in bed (Q4) were independently associated with the diagnosis BPPV. Q2 showed a sensitivity of 43%, and a specificity of 75%; Q4 scored 81% and 68%, respectively. Overall, the way patients perceived their dizziness (vertigo, light-headedness or instability) was not independently associated with the diagnosis BPPV. In younger patients, light-headedness and instability decreased the likelihood of BPPV compared to vertigo.

**Conclusion:** The most reliable predictors for BPPV in patient history are a short duration of the dizziness spell and provocation of dizziness by rolling over in bed. Unlike younger patients, elderly patients with BPPV do not only perceive the dizziness as vertigo, but also as a feeling of instability.

## Introduction

Benign paroxysmal positional vertigo (BPPV) is the most common vestibular type of vertigo, with an estimated incidence of 64 cases per 100,000 people per year and a lifetime prevalence of 2.4% (1, 2). The typical presentation of BPPV consists of recurrent, brief attacks of vertigo generally provoked by changes in head position, such as looking up, bending over, lying down, or rolling over in bed (3–5). However, several alternative presentations are possible. For example, some patients present with complaints of prolonged unsteadiness instead of the typical brief vertigo sensation. This is most common in elderly patients (6).

BPPV is diagnosed by provoking vertigo by positional testing and via observation of typical nystagmus (5). The gold standard for the diagnosis of posterior canal BPPV is the Dix-Hallpike maneuver, whereas horizontal canal BPPV is mostly diagnosed with the supine roll test (5). Besides diagnostic positional maneuvers, patient history is critical for the recognition of BPPV and for classifying the etiology (7, 8). The evaluation of a patient with dizziness can be difficult because of the broad etiology and wide array of questions and tests necessary to come to a diagnosis. It is even more difficult in the geriatric population as increased comorbidity may cause multiple, non-specific symptoms. This may lead to diagnostic delay or leave BPPV unrecognized, which in turn may cause patients to reduce their daily activities and increases the risk of falls (9, 10). Generally, BPPV is well-treatable with canalith repositioning procedures, and as such diagnostic delay could even lead to patients missing out on available treatment. In order to prevent this diagnostic delay and promote early recognition and treatment, it may be helpful to assess which symptoms regarding patient history are the most valuable ones for identifying BPPV in patients presenting with dizziness.

Previous research has identified several items that are useful during history taking of the patient with BPPV, such as the nature of dizziness (i.e., vertigo as opposed to light-headedness), the duration of attacks, and the presence and the frequency of associated symptoms (11–13). Also, the provocative head movement may differ depending on the affected canal. For example, dizziness evoked by looking up could possibly occur more often in patients with posterior canal BPPV than in patients with horizontal canal BPPV.

Another approach that is useful for the early detection of BPPV is the use of predictive models. The purpose of such a model is to predict whether a patient presenting with dizziness has BPPV based on the patient's answers to a set of questions. One of these models is the linear prediction (LP) model developed by Friedland et al., which has shown promising results in the prediction of BPPV (14, 15).

In order to facilitate the diagnosis of BPPV, our primary aim was to identify the questions that are the most valuable for history taking and to validate the predictive LP model of Friedland et al. (14). Secondly, we aimed to assess whether the diagnostic value of these symptoms differed between young and elderly patients and between patients with posterior canal BPPV and horizontal canal BPPV.

## Methods

### Patients and Procedure

This prospective cohort study included all adult patients referred between December 2018 and November 2019 to the Apeldoorn Dizziness Center (ADC), the Netherlands. The ADC is a tertiary referral clinic for patients with dizziness.

All patients were requested to complete a study-specific questionnaire containing six questions about the nature and duration of their dizziness and the positions provoking dizziness (Table 1). This questionnaire, including informed consent form and study information sheet, was sent to patients' home addresses before their visit to the clinic. Patients were asked to select the best suitable option to describe the nature of their dizziness. For the “type of dizziness” item, they could only choose one of the available options. For the questions regarding the duration of dizziness and the positions provoking dizziness, they could only give a Yes or No answer.

**Table 1:** Study-specific questionnaire.

**Question**	**Response options**
Q1—Specify the nature of the main type of dizziness experienced Choose only one option	- Vertigo - Light - headedness - Instability - Other
Q2—Is the duration of the dizziness spell <1 min?	Yes/No
Q3—Does lying down in bed provoke dizziness?	Yes/No
Q4—Does rolling over in bed provoke dizziness?	Yes/No
Q5—Does bending forward provoke dizziness?	Yes/No
Q6—Does looking up provoke dizziness?	Yes/No

The questionnaire was formed based on literature review of diagnostic criteria and questions found indicative of BPPV along with expert opinion (5, 11–13). Because of the goal to quickly and easily recognize BPPV, special interest was placed in concise questions with limited answer possibilities. Questions showing no relevant correlation to BPPV in previous literature were excluded. Questions 3–6 asking about the provocation of the dizziness also had the added goal of elucidating a potential correlation to the type of BPPV (posterior or horizontal).

All patients are subjected to our center's standard examinations for the workup for dizziness, which consist of a routine neuro-otologic examination including positional testing, otoscopy, the video head-impulse test and/or caloric testing, pure-tone audiometry, the hyperventilation provocation test, the postural hypotension test, and completion of the Hospital Anxiety and Depression Scale.

After taking patient history and performing the standard examinations, the ENT surgeon and the neurologist assessed whether the questionnaire and the diagnostic work-up was complete. Patients were excluded if either one was incomplete and the patient was not able or willing to complete the missing questions or tests. Then, they jointly formulated a diagnosis for each patient. The diagnosis of definite BPPV is based on the criteria of the “Consensus Document of the Committee for the Classification of Vestibular Disorders of the Bárány Society” (5). As for the type of BPPV, a torsional-vertical nystagmus with vertigo following the Dix-Hallpike maneuver is classified as posterior canal BPPV, while a horizontal nystagmus with vertigo during the supine roll test is classified as horizontal canal BPPV.

If a patient experienced dizziness without a visible nystagmus, the diagnosis possible BPPV was established (*n* = 6). Patients who experienced BPPV-like symptoms in the past, but had negative provocation maneuvers were diagnosed as historical BPPV (*n* = 82). Both these patient groups were classified as “no BPPV.”

### Statistical Analysis

Continuous variables were described as mean and median, and categorical variables were described as numbers, and percentages.

We assessed the diagnostic value for each of the questions by calculating the odds ratio and its corresponding *p*-value using univariable logistic regression models. Next, the questions eliciting answers that were significantly associated with BPPV (i.e., *p* < 0.05) were entered into a multivariable logistic regression model. For the questions that strongly suggested the diagnosis of BPPV in this model, we calculated the sensitivity and specificity.

Furthermore, we repeated this part of the analysis in subgroups based on the patient's age and on the type of BPPV (horizontal, posterior). Based on the patient's age, we compared if the nature of the dizziness (Q1) differed between patients younger or older than 65 years. The age cut-off was set at 65 because this is a widely accepted cut-off value for seniority. We analyzed differences in provocation of the dizziness (Q3–Q6) by comparing two subgroups based on type of BPPV (horizontal, posterior).

For validation of the diagnostic LP model for BPPV of Friedland et al., we used our study-specific questionnaire data to create an LP value for each patient. The model of Friedland et al. was completed by substituting the variable with a “1” if it was present or a “0” if it was not present. “Lying Down or Rolling Over” was classified as “1” if a patient reported provocation of dizziness by lying down in bed or rolling over (Q3 or Q4). “Vertigo” was set as “1” if the nature of the dizziness was classified as vertigo (Q1).

(1) $$\begin{array}{l} \text{LP}=-2.19\;+\;1.87\;\times \;\left(\text{Lying Down or Rolling Over}\right)\\ \;+0.92\;\times \;\left(\text{Vertigo}\right)-0.98\;\\ \;\times \;\left(\text{LOS}:\text{Minutes to Hours}\right)-1.11\;\\ \;\times \;\left(\text{LOS}:\text{Days}\right)-1.84\;\times \;\left(\text{Vertigo}\right)\\ \;\times \;\left(\text{LOS}:\text{Days to Weeks}\right) \end{array}$$

The LP value was then converted to an estimated probability:

(2) $$\begin{array}{l} \mathrm{Pr}\left(BPPV\right)=\;\frac{e^{LP}}{1+\;e^{LP}} \end{array}$$

In our study-specific questionnaire, we distinguished between duration of more or <1 min (Q2), and as such we did not have access to duration data to the same extent as Friedland et al. whose formula splits the length of duration (LOS) into separate entities (minutes to hours, days, days to weeks). Hence, we classified all LOS variables as “0” if our patients reported dizziness lasting shorter than 1 min. Dizziness lasting more than 1 min was categorized by chance as “LOS: Minutes to Hours,” or “LOS: Days” in a 50:50 distribution, except for dizziness perceived as vertigo and lasting longer than 1 min, which was categorized as “LOS: Days to Weeks.”

The cut-off value ≥0.2 was used as confirmation of BPPV, based on a previous study by Friedland et al. (14). Based on these LP values, a confusion matrix was produced, and the sensitivity and specificity of the model were calculated by means of a receiver operating characteristic (ROC) curve.

### Ethical Considerations

The study was conducted in accordance with the ethical standards laid down in the 1964 Declaration of Helsinki and its later amendments up to 2013 (16) and was approved by the Local Review Board of Gelre Hospital. Written informed consent for participation in the study was obtained from all participants.

## Results

A total of 885 patients were included in the study, 113 of whom (13%) were diagnosed with BPPV. The mean age of the population was 57 years (SD 16.8, min/max 18–92), and 568 (64%) were female (Table 2). The mean age of patients diagnosed with BPPV was significantly higher than the mean age of patients without BPPV [62 years (SD 16) vs. 57 years (SD 17), *p* < 0.01], and the proportion of female patients was significantly higher in the BPPV group [73% (*n* = 82) vs. 63% (*n* = 486), *p* < 0.05]. Out of 113 patients diagnosed with BPPV, 101 (89%) patients had posterior canal BPPV, 11 patients were diagnosed with horizontal canal BPPV, and one patient suffered from anterior canal BPPV. The patient with anterior canal BPPV was excluded from analysis because multinomial logistic regression cannot be applied to a group of one.

**Table 2:** Patient demographics.

		**BPPV** ***n* (% in group)**	**No BPPV** ***n* (% in group)**	**Total** ***n* (% of total)**	***p*-Value**
Number		113 (13%)	772 (87%)	885	
Age	*Mean (years)*	61.9	56.7	57.4	<0.01
	*Elderly (≥65 years)*	55 (49%)	269 (35%)	324 (37%)	
Gender	*Female*	82 (77%)	486 (63%)	568 (64%)	<0.05
Type of BPPV	*Posterior*	101 (89%)	NA	NA	
	*Horizontal*	11 (10%)	NA	NA	
	*Anterior*	1 (1%)	NA	NA	

*BPPV, benign paroxysmal positional vertigo; NA, not applicable*.

### Uni- and Multivariable Analysis of Study-Specific Questionnaire

Univariable analysis showed a correlation with all questions and the diagnosis of BPPV, except for the way patients perceived their dizziness (vertigo or instability, Q1) (Table 3). While the presence of light-headedness rather than vertigo reduced the chance of BPPV being present [OR: 0.45 (95% CI 0.24– 0.87), *p* = 0.02], dizziness experienced either as instability or vertigo was not significantly associated with a BPPV diagnosis [OR: 0.67 (95% CI 0.40–1.1), *p* = 0.10]. A total of 91 (81%) patients with BPPV reported dizziness provoked by turning over in bed, compared to 243 (32%) patients without BPPV. This resulted in an odds ratio of 8.94 (95% CI 5.5–14.6, *p* < 0.01).

**Table 3:** Univariable and multivariable analysis for association between study-specific questions and diagnosis of BPPV (BPPV, *n* = 113; no BPPV, *n* = 772).

**Question**			**Univariable analysis**			**Multivariable analysis**	
	**Missing**	**Answer**	***n* (% of answer)**	**OR (95% CI)**	***p*-Value**	**OR (95% CI)**	***p*-Value**
Q1—Type of dizziness	61	*Vertigo*	50 (16%)	1.0	–	1.0	–
		*Light-headedness*	13 (8%)	0.45 (0.2–0.9)	0.02	0.70 (0.35–1.40)	0.32
		*Instability*	32 (11%)	0.67 (0.4–1.1)	0.10	1.2 (0.69–2.0)	0.54
		*Unspecified*	8 (12%)	0.71 (0.3–1.6)	0.40	1.06 (0.45–2.51)	0.90
Q2–Duration <1 min.	1	*No*	64 (10%)	1.0	–	1.0	–
		*Yes*	49 (20%)	2.26 (1.5–3.4)	<0.0001	1.77 (1.11–2.83)	0.02
Q3–Provoked by lying down in bed	2	*No*	50 (8%)	1.0	–	1.0	–
		*Yes*	63 (28%)	4.80 (3.2–7.2)	<0.0001	1.48 (0.87–2.50)	0.14
Q4–Provoked by turning over in bed	4	*No*	22 (4%)	1.0	–	1.0	–
		*Yes*	91 (27%)	8.94 (5.5–14.6)	<0.0001	6.01 (3.27–11.0)	<0.0001
Q5–Provoked by looking up	1	*No*	35 (9%)	1.0	–	1.0	–
		*Yes*	78 (16%)	1.94 (1.3–3.0)	0.002	1.064 (0.64–1.78)	0.81
Q6–Provoked by bending over	1	*No*	25 (7%)	1.0	–	1.0	–
		*Yes*	88 (17%)	2.85 (1.8–4.5)	<0.0001	1.26 (0.71–2.22)	0.43

*OR, odds-ratio; CI, confidence interval*.

Multivariable analysis demonstrated that a duration of dizziness spells <1 min (Q2) [OR: 1.8 (95% CI 1.1 to 2.8), *p* = 0.02] and dizziness provoked by turning over in bed (Q4) [OR 6.0 (95% CI 3.2–11.0), *p* < 0.01] were independently associated with the diagnosis of BPPV (Table 3). The duration of the dizziness spell <1 min (Q2) had a sensitivity of 43% and a specificity of 75%. For dizziness provoked by turning over in bed (Q4), these percentages were 81 and 68%, respectively.

The way patients perceived their dizziness (vertigo, light-headedness, or instability) was not independently associated with the diagnosis BPPV.

### Subgroup Analysis

We observed no significant differences in the manner of provocation (Q3–Q6) between the patients with posterior canal BPPV and the ones with horizontal canal BPPV.

In a subgroup analysis comparing the older to the younger patients, 324 (37%) patients were assigned to the elderly group (≥65 years). Of these patients, 55 (49%) were diagnosed with BPPV (Table 2). In the group of elderly patients, we did not find an association between the perceived type of dizziness and the occurrence of BPPV, while for the younger patients, BPPV was negatively associated with light-headedness and instability compared to vertigo [OR: 0.37 (95% CI 0.16–0.87), *p* = 0.02; OR: 0.36 (95% CI 0.15–0.84), *p* = 0.02] (Table 4).

**Table 4:** Subgroup analysis for elderly and younger patients regarding perceived type of dizziness and diagnosis of BPPV.

	**Missing**	**Perceived type of dizziness**	***n* (% of answer)**	**OR (95% CI)**	***p*-Value**
Elderly (≥65 years)	30	*Vertigo*	16 (22%)	1.0	–
		*Light-headedness*	6 (14%)	0.60 (0.2–1.7)	0.34
		*Instability*	25 (16%)	0.68 (0.3–1.4)	0.27
		*Other*	3 (17%)	0.73 (0.2–2.8)	0.64
Younger	31	*Vertigo*	34 (14%)	1.0	–
		*Light-headedness*	7 (6%)	0.37 (0.2–0.9)	0.02
		*Instability*	7 (6%)	0.36 (0.2–0.8)	0.02
		*Other*	5 (10%)	0.68 (0.3–1.8)	0.45

*OR, odds-ratio; CI, confidence interval*.

### Validation of LP Model

We validated the LP model for BPPV of Friedland et al. (14). Using the cut-off value of ≥0.2 for the LP value, the sensitivity was 83% and the specificity 66%. The ROC-curve showed that the AUC of this model was 0.76 (Supplementary Figure 1). Using the ROC-curve, we found that lowering the cut-off point to ≥0.15 changed the sensitivity to 66% and the specificity to 83%.

## Discussion

In this prospective cohort study, we determined the diagnostic value of several questions for the recognition of BPPV in patients with vestibular complaints. This would enable easy and fast assessment of patients presenting with dizziness, which can be valuable for otolaryngologists and neurologists, physicians working in emergency rooms, and general practitioners working in primary care. Early recognition of BPPV could reduce diagnostic delay and could prevent physicians from missing the diagnosis altogether, which puts the patient at an increased risk of falling, impairment of daily activities, and missing out on treatment.

Two symptoms showed a strong and independent association with BPPV: the specific trigger of rolling over in bed, and a dizziness spell with a duration of <1 min. These results are in line with several previous studies, which also found that these symptoms had a positive association with BPPV (11, 13, 14, 17–21).

We found that whether patients perceived their dizziness as vertigo or instability was not a predictor of BPPV, whereas a perception of light-headedness clearly was a negative predictor for a diagnosis of BPPV. In subgroup analysis, both light-headedness and instability in younger patients were negatively associated with the diagnosis of BPPV (*p* < 0.05). This result is in accordance with results reported by Batuecas-Caletrio et al. and Piker et al. who showed that elderly patients with BPPV present less frequently with the classic vertigo sensation (6, 22). This could be explained by a decreased sensitivity of the otolithic organ due to otoconial degeneration (23, 24).

The question concerning the type of perceived dizziness had a substantially higher number of missing entries (*n* = 61, 7%) than the other study-specific questions (*n* = 1–4, 0.1–0.5%). Patients who did not answer this question either could not characterize their dizziness as one of the possible options or experienced multiple types of dizziness. The high number of missing entries shows that many patients find it difficult to describe the nature of their dizziness. This finding is consistent with the literature. Newman-Toker et al. found that patients were inconsistent and unreliable in their answers when having to pick a single type of dizziness (25). Seventy-nine percent of the patients picked more than one answer when given that option. In comparison, questions related to symptom duration and dizziness triggers were answered more consistently, and Newman-Toker et al. concluded that these questions would prove more useful for diagnosing BPPV (25). A possible explanation for the difficulty patients have in characterizing their dizziness is lack of familiarity with dizziness-related symptoms, as well as the fact that the duration of symptoms often is too short to assess the quality (26).

Combining the high rate of missing or inconsistent answers regarding the nature of the dizziness and our finding that there is no association between the type of dizziness and the presence of BPPV, we propose that the nature of dizziness should play a less prominent role in the diagnostic work-up of BPPV. Instead, the primary questions should be the ones focusing on triggers and on symptom duration. This change in priority will require a change in procedure and in education, as it has been shown previously that the majority of physicians endorse a main role for the nature of dizziness in determining of the etiology of dizziness (27).

We found no association between the type of head movement provoking dizziness and the affected semicircular canal of the vestibular organ. A possible explanation of this result could be the small number of patients diagnosed with horizontal canal BPPV in our study population (*n* = 11). To our knowledge, only one article has previously examined this possible association (28). The authors investigated whether the trigger for the dizziness could predict which semicircular canal is affected, but they did not find an association between the two phenomena. Further research is required to elucidate the apparent discrepancy between known pathophysiology and clinical practice.

Besides establishing which questions should be prioritized in the diagnosis of BPPV, we also validated the predictive model of Friedland et al. (14) with the data of our study-specific questionnaire. Using a cut-off of ≥0.2 for the LP value, we found that the model had a high sensitivity of 82% and a specificity of 66% with an AUC of 0.76. Our findings are almost identical to the cross-validation by Friedland et al. who found a sensitivity of 79% and specificity of 65%, with an AUC of 0.76 (14). Changing the cut-off point to 0.15 in our population changed the sensitivity to 66% and the specificity to 83%. This cut-off point could be more useful for ruling out the suspected diagnosis of BPPV, and it would prevent patients with a negative result from undergoing unnecessary diagnostic tests. Comparing the diagnostic power of the model to the two predictors with the strongest association with BPPV (Q2—duration of the spell <1 min, Q4—provoked by rolling over in bed), the model performs similarly to “provocation by rolling over in bed,” but does not seem to add any diagnostic value over the use of single questions. However, the limitations of this result should be kept in mind, as we did not have all the data necessary for the length of spell variables (minutes to hours, days, days to weeks). The assumption that all vertiginous dizziness lasting longer than 1 min counts as a positive interaction term [“1.84 × (Vertigo) × (LOS: Days to Hours)”] most likely results in an overestimation of this term. This overestimation results in a lower LP value and a structurally lower Pr(BPPV), resulting in fewer patients diagnosed with BPPV by means of this formula than is actually the case. It is therefore expected that our assumption results in an underestimation of the sensitivity found for the LP model.

A potential limitation of our study lies in the fact that our results are not suited to be extrapolated to first-line medical care as provided by emergency departments and general physicians. Because the data were gathered in a tertiary center, there is a risk of selection bias, and the patient population most likely has different characteristics than a non-preselected population. However, similar results have been found in studies set in a general medical department and in a secondary emergency hospital (11, 13).

Another limitation concerns the generalization of the two questions which were found to associate most strongly with a diagnosis of BPPV. Because these questions were part of a set of only six questions instead of a broader set, they might have erroneously been marked as correlated to BPPV. However, considering the findings compare favorably with previously published literature (11, 13, 14, 17–21), the influence of this limitation is most likely minor.

In conclusion, a strong, independent association exists between BPPV and the duration of a dizziness spell and the trigger situation of rolling over in bed. Interestingly, the nature of the dizziness was only of diagnostic importance in younger patients. We did not find an association between different types of provocative head movements and the affected semicircular canal. The predictive model of Friedland proved to perform well for confirming the suspected diagnosis of BPPV, but did not add diagnostic value compared to dizziness provocation by rolling over in bed.

## Data Availability Statement

The original contributions presented in the study are included in the article/Supplementary Materials, further inquiries can be directed to the corresponding author/s.

## Ethics Statement

The studies involving human participants were reviewed and approved by the Local Review Board of Gelre Hospital. The patients/participants provided their written informed consent to participate in this study.

## Author Contributions

TB: research idea. VD and BM: designed the study. VD: analyzed the data and wrote the manuscript. TB, BM, and TS: revised the manuscript. All authors contributed to the article and approved the submitted version. They confirm that questions related to the accuracy of the work were adequately discussed and resolved.

## Conflict of Interest

The authors declare that the research was conducted in the absence of any commercial or financial relationships that could be construed as a potential conflict of interest.
